# Synergistic impact of diabetes and cognitive impairment on all-cause and cause-specific mortality in Chinese older adults: A prospective population-based cohort study

**DOI:** 10.3389/fendo.2022.997260

**Published:** 2022-11-14

**Authors:** Zhiqiang Li, Shengshu Wang, Shaohua Liu, Xinran Gong, Yanding Wang, Di Wu, Meitao Yang, Rongrong Li, Haowei Li, Xuehang Li, Shimin Chen, Ruizhong Jia, Jinpeng Guo, Jianhua Wang, Miao Liu, Yao He, Yong Wang

**Affiliations:** ^1^ School of Public Health, China Medical University, Shenyang, China; ^2^ Center for Disease Control and Prevention of Chinese People’s Liberation Army, Beijing, China; ^3^ Beijing Key Laboratory of Aging and Geriatrics, Institute of Geriatrics, National Clinical Research Center for Geriatrics Diseases, Second Medical Center of Chinese People's Liberation Army (PLA) General Hospital, Beijing, China; ^4^ Department of Healthcare, Agency for Offices Administration, Central Military Commission, People’s Republic of China, Beijing, China; ^5^ Department of Epidemiology and Statistics, Graduate School of Chinese People's Liberation Army (PLA) General Hospital, Beijing, China; ^6^ Department of Epidemiology, State Key Laboratory of Kidney Diseases, Chinese People's Liberation Army (PLA) General Hospital, Beijing, China

**Keywords:** diabetes mellitus, cognitive impairment, old adults, cause-specific mortality, Chinese

## Abstract

**Background:**

Diabetes mellitus (DM) or cognitive impairment (CI) is known to be strongly associated with mortality. DM commonly coexists with CI and proportionally increases with age. However, little is known about the combined effect of cognitive function and diabetes on mortality. This study aimed to evaluate the combined effects of DM and CI on all-cause and cause-specific mortality in Chinese older adults.

**Methods:**

This prospective population-based cohort study was based on the Beijing Elderly Comprehensive Health Cohort Study. A total of 4,499 older adults were included. Cox’s proportional hazard models were utilized to calculate the effect of DM and CI on all-cause, cardiovascular disease (CVD) mortality and cancer mortality, and a multiplicative term was introduced to study a potential interaction between DM and CI on outcomes.

**Results:**

During a median follow-up of 6.8 years (ranging from 6.6 to 11.7 years), 667 (14.8%) participants died from all causes, 292 from CVD, and 215 from cancer. In the fully adjusted model, participants with coexisting DM and CI had the highest risk of all-cause mortality [hazard ratios (HRs), 3.08; 95% confidence intervals (CIs), 2.30,4.11] and CVD mortality (HRs, 3.85; 95% CIs, 2.60,5.71) compared with individuals with normal cognition and non-DM. We also found a multiplicative interaction between DM and CI in respect to all-cause (HRs, 2.46; 95% CI, 1.87,3.22) and CVD mortality (HRs, 3.15 95% CI, 2.19,4.55). In the diabetic population, CI was associated with an increased risk of all-cause mortality (HRs, 2.09; 95% CIs, 1.51,2.89) and CVD mortality (HRs, 3.16; 95% CIs, 2.02,5.05) compared with the normal cognition group. Restricted cubic spline revealed a linear inverse association between Mini-Mental State Examination (MMSE) score and all-cause, CVD mortality in the total sample and participants without diabetes. However, a nearly reverse J association was observed between MMSE and mortality from all causes and CVD in the diabetes group.

**Conclusion:**

The findings highlighted that cognitive impairment concomitant with diabetes further increases the risk of mortality. In addition to strengthening routine screening of cognitive functioning in older adults with early-stage diabetes, more extensive assessment of prognostic risks has high clinical value for developing comprehensive treatment plans.

## Introduction

Diabetes mellitus (DM) is increasing globally in both prevalence and population size as a common metabolic disease, especially in China (1). Epidemiological studies have reported that DM is associated with an increased risk of cause-specific mortality (2–4). However, much less information is available in China. Additionally, there are few studies on the impact of DM on mortality risk for Chinese older adults living in the community, which deserves further study.

Cognitive impairment (CI) is a highly prevalent mental disorder in older adults and is considered an intermediate transitional stage between cognitively normal and dementia (5). China is one of the countries with the fastest aging worldwide, the prevalence of CI in older adults is increasing year by year, and the number of people affected is expected to exceed 140 million by 2050 (6). A recent meta-analysis, which explored the association between CI and mortality, observed that CI detected by the Mini-Mental State Examination (MMSE) is associated with an elevated risk of all-cause mortality (7). Some studies in Western populations have also shown that individuals with CI increase cause-specific mortality (8–10). However, the link between cognitive function and cause-specific mortality risk in Chinese older adults is still lacking.

Both DM and CI are long-term disabling conditions. They share a common pathological mechanism and coexist in people older than 60 years (11). Interestingly, DM is a known potentially modifiable risk factor for CI (12). There is evidence that CI is a serious complication of DM that adversely affects the brain of patients with DM; individuals with DM are twice as likely to develop dementia as those without DM (13, 14). Meanwhile, CI, as a high-risk group for dementia, has 10%–15% of patients with CI progress to dementia per year, and DM can accelerate the transformation rate by 1.5–3.0 times (15). However, the control of DM is no longer a traditional treatment but a systematic management (16, 17). CI leads to self-management difficulties and increases the risk of DM and its complications (11, 14, 18). Therefore, DM and CI form a two-way vicious circle.

In addition to the vascular complications of DM itself or the risk of mortality caused by blood glucose fluctuations, CI is an important prognostic factor for the elderly with DM, and the impact on the prognosis when combined with DM is still unclear. Although the current expert consensus on diabetic cognitive dysfunction in the Chinese population has been published (19), a comprehensive examination of their associations with long-term outcomes is not available. Hence, we assessed the combined effect and multiplicative interaction of these two conditions on all-cause and cause-specific mortality in the Beijing Elderly Comprehensive Health Cohort Study (BECHCS).

## Methods

### Study design and population

The BECHCS is a prospective cohort study, which was based on urban and rural areas of older adults. Study design and detailed information on the BECHCS have been reported elsewhere (20, 21). Participants who met the following criteria were included in the BECHCS. Inclusion criteria were the following: (1) aged 60 years or above who had lived in the Wanshou Road Community of Haidian District and Miyun county (≥1 year) in Beijing, (2) could understand and cooperate to complete the study evaluation, and (3) willing to take part in the physical examination and biological sample collection. The exclusion criteria were as follows: (1) those with serious diseases or functional disorders, (2) those who were unable to participate in the physical examination and evaluation items, and (3) those with suspected dementia (MMSE score <6). A total of 4,834 elderly residents were recruited in the baseline survey. Follow-up was conducted every 2–3 years, with follow-up information focusing on the participant’s survival status, and the time, place, and cause of death. In our statistical analysis, we excluded 335 participants for missing data; finally, the resulting cohort included 4,449 participants.

Briefly, this cohort of the elderly population was based on a two-stage stratified random-clustering sampling method between 2010 and 2014. Two districts representing the urban and rural areas of Beijing were selected to constitute the sample. These participants accounted for about 10% of total elderly residents. The Ethics Committee of Chinese PLA General Hospital approved this study (Ethics Number: S2021-327-01). All participants gave written informed consent. The results of our article will be provided regarding the Strengthening the Reporting of Observational Studies in Epidemiology (STROBE) reporting guidelines.

### Diabetes and cognitive impairment

Blood samples of all participants were collected after an overnight fast of at least 10 h. Participants were diagnosed with DM if they met the following conditions: (1) self-reported diabetes diagnosis, (2) using a prescription of oral glucose-lowering medication regularly, and (3) newly diagnosed diabetes. According to the American Diabetes Association (ADA) criteria (22), a self-reported diabetes diagnosis is define as a diagnosis determined previously by professional healthcare institutions, and a newly diagnosed diabetes is defined as those with fasting plasma glucose (FPG) level ≥7.0 mmol/L (126 mg/dl) and glycated hemoglobin (HbA1c) ≥6.5% (48 mmol/mol) among participants without self-reported diabetes. Total DM included both previously diagnosed diabetes and newly diagnosed diabetes.

The MMSE is a sensitive evaluation scale, which is adapted from the scale developed by Folstein and used widely to evaluate cognitive function (23). The MMSE score ranges from 0 to 30; the lower the score, the more severe cognitive problems. This study used the Chinese version of MMSE, which has been validated in the Chinese population for screening test (24, 25) Consistent with the method described in the previous study (21), CI was defined using education-adjusted MMSE cutoff points (MSE <17, uneducated participants; MMSE <20, participants with primary education level; MMSE <24, participants with over 6 education years).

### Ascertainment of mortality

The data on vital status were obtained from the Chinese Center for Disease Control and Prevention, and it was verified by the medical insurance system or public security department. The follow-up time was calculated from the date of entering the cohort to the date of death or censoring (March 2021). The International Statistical Classification of Diseases and Related Health Problems, 10th Revision (ICD-10), was used to classify the specific cause of death. Causes were defined as follows: CVD (I00–I99) and cancer mortality (C00–C97) have specific codes.

### Covariates

Covariates included demographic information [age (continuous), gender (male/female), residence (urban/rural), education years (0,1–6,>6), marital status (married/widowed/others)], lifestyle information [smoking status (current/former/never smoker), alcohol consumption (current/former/never drinker), exercise (<0.5 h/day vs. ≥0.5 h/day)], and chronic diseases [hypertension (yes/no), cardiovascular disease (yes/no), dyslipidemia (yes/no), chronic obstructive pulmonary disease (COPD) (yes/no), tumor (yes/no)]; all those diseases were identified according to self-reporters. Anthropometric variables [systolic blood pressure (SBP), diastolic blood pressure (DBP), and waist circumference (WC)] and biological indicators [FPG, HbA1c, triglyceride (TG), total cholesterol (TC), and uric acid] were also included. Body mass index (BMI) was calculated in kilograms divided by the square of height in meters (kg/m^2^)].

Serum samples in all participants were drawn and frozen at −20°C within 2 h of collection and shipped by air in dry ice and sent to the central certified laboratory of Chinese PLA General Hospital. The levels of FPG and HbA1c were measured by an enzymatic method using the Roche Cobas 8000 automatic biochemical analyzer (Switzerland). TG and TC were measured using the Hitachi 7080 automatic Biochemical analyzer (Japan), Uric acid was measured by Changchun Dirui H-800 (China). All biochemical analyses were carried out using standardized laboratory methods.

### Statistical analysis

Participants were categorized into four-level joint groups (normal cognition and non-DM, normal cognition and DM, cognitive impairment and non-DM, and cognitive impairment and DM) according to different combinations of DM (yes or no) and CI (yes or no). Baseline characteristics of participants were described by the four groups. Data were presented as numbers (percentages) for categorical variables and as means (standard deviations, SD) or medians (interquartile range, IQR) for continuous variables. The differences between groups were applied to ANOVA or Kruskal–Wallis test for continuous variables and χ^2^ test for categorical variables. The R package (“forestplot,” “ggplot2,” “survival,” and “rms”) (3.6.1 version, R Foundation for Statistical Computing, Vienna, Austria) and SPSS (20.0, Chicago, IL) were used to analyze data. All statistical tests were two-tailed, and p-values <0.05 were considered statistically significant.

Association between DM, CI, and the risk of all-cause, cause-specific mortality was analyzed by fitting the four types of Cox proportional hazards models. Hazard ratios (HRs) and 95% confidence intervals (CIs) were reported. Model 1 was an unadjusted model, and further adjusted for gender and age in model 2, then further adjusted for residence, education years, marriage, alcohol drinking, smoking status, exercise, and BMI in model 3. In model 4 (the fully adjusted model), we further adjusted for WC, TC, TG, uric acid, and chronic diseases (hypertension, coronary disease, dyslipidemia, COPD, and tumor). The proportional hazard assumption was assessed based on Schoenfeld residuals, and it no violation had been found. When the causes of CVD and cancer were simultaneously modeled as different events, the competing risk model was conducted to compare the association with CI and DM among causes of death (26). We also fitted a stratified model to evaluate the association of CI on the outcomes within strata of DM, and a cross-product interaction term of DM and CI (‘DM’×’CI’) was added to those models above to evaluate whether there was a multiplicative interaction effect between cognitive function status and the presence of diabetes on mortality. Restricted cubic splines with three knots at 17, 21, and 24 points (MMSE) were used to analyze the association of MMSE with mortality in total study participants and participants with or without DM based on the fully adjusted Cox proportional hazards model. The likelihood ratio test was used to test for potential non-linearity (25, 27).

A comparison of the survival curve among the four-level joint groups of DM/CI was conducted by the Kaplan–Meier and the log-rank test. To explore the important confounding factors influencing the link between the four groups and mortality, stratified analyses were conducted by age (60–79 and ≥80 years), gender (male/female), and residence (urban/rural). The interactions between baseline four-level joint groups and the aforementioned subgroup variables were tested to evaluate whether the combined effect was similar in different subgroups.

To further test the stability of results, several sensitivity analyses were performed: (1) all participants who died within 2 years of follow-up were excluded; (2) participants with four kinds of self-reported diseases (hypertension, coronary disease, dyslipidemia, and tumor) were excluded; (3) participants with prediabetes were excluded (28); (4) participants with newly diagnosed DM were excluded; (5) additionally adjusting was performed for family history of hypertension, diabetes, and coronary disease, which are important factors related to DM, CI, and mortality; (6) MMSE <23 was used to define CI (29); (7) CI was defined as follows (29)—for no formal education participants, MMSE <18; for participants with elementary education, MMSE < 21; and for participants with more than 7 education years, MMSE <25; (8) a secondary analysis of the primary outcome was conducted after use of new criteria for the severity of CI (MMSE ≥24, 18–23, and < 18 were used to define normal cognition, mild CI, and severe CI).

## Results

### Baseline characteristics

Among the 4,499 participants [mean(SD) age, 70.3 (6.7) years; 2,684 (59.7%) female] included in analyses of diabetes and cognitive function status, the average MMSE score was 24.31(SD, 5.45). In addition, 1,076 individuals (23.9%) had DM at baseline, and 763 older adults had CI, accounting for 17% of the sample. Compared with participants without CI and DM, participants who had both DM and CI were more likely to be older, female, live in rural regions, widowed, have a lower level of receiving education, and have less physical activity (<0.5 h/day). There were differences in the prevalence of hypertension, coronary disease, dyslipidemia, and tumor between the groups. Among patients with CI, having diabetes was also associated with lower scores of MMSE and higher levels of FPG, HbA1c, BMI, WC, TC, and TG (**Table 1**).

**Table 1:** Baseline characteristics of participants by diabetes and cognition categories.

Characteristics	Normal cognition and non-DM	Normal cognition and DM	CI and non-DM	CI and DM	Overall	*p-*value
No. of participants	**2,836**	**900**	**587**	**176**	**4,499**	
Age, mean (SD)	69.68 (6.56)	70.16 (6.03)	72.78 (7.68)	72.20 (7.10)	70.28 (6.73)	<0.001
MMSE, mean (SD)	26.08 (3.47)	26.09 (3.45)	15.57 (5.12)	15.91 (5.07)	24.31 (5.45)	<0.001
FPG, median (IQR)	5.37 (5.03,5.76)	7.31 (6.42,8.62)	5.28 (4.88,5.68)	7.33 (6.58,9.27)	5.55 (5.11,6.26)	<0.001
HbA1c, median (IQR)	5.60 (5.40,5.90)	6.80 (6.30,7.70)	5.60 (5.40,5.90)	6.70 (6.20,7.80)	5.80 (5.40,6.10)	<0.001
BMI, median (IQR)	24.44 (22.64,26.37)	25.08 (23.44,27.18)	24.56 (22.60,25.39)	25.07 (23.56,27.25)	24.69 (22.86,26.4)	<0.001
WC, median (IQR)	88.00 (83.00,93.00)	90.00 (86.00,96.00)	88.00 (84.00,90.00)	90.00 (86.00,95.50)	89.00 (84.00,93.0)	<0.001
TC, median (IQR)	4.89 (4.26,5.59)	4.96 (4.34,5.71)	4.79 (4.18,5.51)	4.86 (4.18,5.58)	4.89 (4.25,5.59)	0.019
TG, median (IQR)	1.28 (0.93,1.79)	1.56 (1.07,2.24)	1.21 (0.87,1.67)	1.51 (1.11,2.08)	1.32 (0.95,1.85)	<0.001
Uric acid, median (IQR)	294.00 (245.00,351.00)	295.40 (246.40,353.00)	281.16 (226.85,338.27)	276.20 (228.90,338.10)	291.02 (241.80,349.00)	0.001
Female, n (%)	1,599 (56.4)	580 (64.4)	378 (64.4)	127 (72.2)	2,684 (59.7)	<0.001
Residence, n (%) Urban Rural	1,389 (49.0) 1,447 (51.0)	533 (59.2) 367 (40.8)	126 (21.5) 461 (78.5)	54 (30.7) 122 (69.3)	2,102 (46.7) 2,397 (53.3)	
Marriage, n (%) Married Widowed Others	1,180 (41.6) 1,424 (50.2) 232 (8.2)	455 (50.6) 363 (40.3) 82 (9.1)	96 (16.4) 448 (76.3) 43 (7.3)	43 (24.4) 118 (67.0) 15 (8.5)	1,774 (39.4) 2,353 (52.3) 372 (8.3)	<0.001
Education, years n (%) 0 1-6 >6	656 (23.1) 909 (32.1) 1,271 (44.8)	211 (23.4) 262 (29.1) 427 (47.4)	264 (45.0) 196 (33.4) 127 (21.6)	68 (38.6) 56 (31.8) 52 (29.5)	1,199 (26.7) 1,423 (31.6) 1,877 (41.7)	<0.001
Alcohol drinking, n (%) Never Former Current	1,715 (60.5) 142 (5.0) 979 (34.5)	621 (69.0) 58 (6.4) 221 (24.6)	334 (56.9) 41 (7.0) 212 (36.1)	121 (68.8) 9 (5.1) 46 (26.1)	2,791 (62.0) 250 (5.6) 1,458 (32.4)	<0.001
Smoking status, n (%) Never Former Current	1,939 (68.4) 378 (13.3) 519 (18.3)	638 (70.9) 135 (15.0) 127 (14.1)	417 (71.0) 61 (10.4) 109 (18.6)	133 (75.6) 20 (11.4) 23 (13.1)	3,127 (69.5) 594 (13.2) 778 (17.3)	0.008
Exercise <0.5 h/day ≥0.5 h/day	899 (31.7) 1,937 (68.3)	245 (27.2) 655 (72.8)	288 (49.1) 299 (50.9)	72 (40.9) 104 (59.1)	1,504 (33.4) 2,995 (66.6)	<0.001
Hypertension, n (%) Yes No	1,783 (62.9) 1,053 (37.1)	650 (72.2) 250 (27.8)	409 (69.7) 178 (30.3)	126 (71.6) 50 (28.4)	2,968 (66.0) 1,531 (34.0)	<0.001
Coronary disease, n (%) Yes No	544 (19.2) 2,292 (80.8)	246 (27.3) 654 (72.7)	100 (17.0) 487 (83.0)	48 (27.3) 128 (72.7)	938 (20.8) 3,561 (79.2)	<0.001
Dyslipidemia, n (%) Yes No	486 (17.1) 2,350 (82.9)	239 (26.6) 661 (73.4)	44 (7.5) 534 (92.5)	24 (13.6) 152 (86.4)	793 (17.6) 3,706 (82.4)	<0.001
COPD, n (%) Yes No	228 (8.0) 2,608 (92.0)	80 (8.9) 820 (91.1%	34 (5.8) 553 (94.2)	9 (5.1) 167 (94.9)	351 (7.8) 4,148 (92.2)	0.080
Tumor, n (%) Yes No	183 (6.5) 2,653 (93.5)	69 (7.7) 831 (92.3)	20 (3.4) 567 (96.6)	7 (4.0) 169 (96.0)	279 (6.2) 4,220 (93.8)	0.005

SD, standard deviation; CI, cognitive impairment; DM, diabetes mellitus; WC, waist circumference; FPG, fasting plasma glucose; HbA1c, glycated hemoglobin; MMSE, Mini-Mental State Examination. Chronic diseases (hypertension, cardiovascular disease, dyslipidemia, COPD, tumor) that coexist in the same older person.

### Diabetes and cognitive status, and mortality

The survival curves showed distinct outcome trajectories for the four categories stratified by diabetes and cognitive function (log-rank p<0.001, **Supplementary eFigure S1**). The median follow-up period of all-cause mortality in the four CI/DM groups was 6.8, 11.0, 6.7, and 6.7, respectively. During a median follow-up of 6.8 years, 667 participants died, including 292 CVD deaths and 215 cancer deaths.

The combined associations between DM, CI, and mortality are displayed in **Table 2**. The group of normal cognition and non-DM was used as the reference. In the fully adjusted model, we found that participants who had CI and DM had the highest risk of all-cause mortality (HRs, 3.08; 95% CIs, 2.30,4.11) and CVD mortality (HRs, 3.85; 95% CIs, 2.60,5.71), compared with the reference group. Furthermore, participants with only DM had a higher risk of all-cause mortality (HRs, 1.42; 95% CIs, 1.16,1.72) and cancer mortality (HRs, 1.47; 95%CIs, 1.05,1.72); participants with only CI had a higher risk of all-cause mortality (HRs, 1.39; 95% CIs, 1.09,1.74), CVD mortality (HRs, 1.53; 95% CIs, 1.09,2.15) and cancer mortality (HRs, 1.49; 95%CIs, 1.00,2.22), whereas those who had CI and DM had no significant increase in the risk of cancer mortality. After using the competing risk analysis, the association of mortality with CI and DM did not statistically differ among causes of death (p = 0.64).

**Table 2:** Hazard ratios for the combined associations of DM and CI with all-cause and cause-specific mortality.

	Normal cognition and non-DM	Normal cognition and DM	CI and non-DM	CI and DM
Participants, no.	2,836	900	587	176
**All-cause mortality**				
Death, No.	346	156	108	57
Model 1^a^	1 (ref)	1.34 (1.11,1.62)*	2.09 (1.68,2.60)*	3.58 (2.71,4.75)*
Model 2^b^	1 (ref)	1.39 (1.15,1.68)*	1.63 (1.30,2.03)*	3.21 (2.42,4.26)*
Model 3^c^	1 (ref)	1.42 (1.17,1.72)*	1.37 (1.08,1.72)**	3.12 (2.34,4.16)*
Model 4^d^	1 (ref)	1.42 (1.16,1.72)*	1.39 (1.09,1.74)**	3.08 (2.30,4.11)*
**CVD mortality**				
Death, no.	144	63	52	33
Model 1^a^	1 (ref)	1.30 (0.97,1.75)	2.52 (1.83,3.47)	5.13 (3.51,7.50)*
Model 2^b^	1 (ref)	1.35 (1.01,1.82)**	1.79 (1.29,2.49)*	4.45 (3.05,6.51)*
Model 3^c^	1 (ref)	1.38 (1.02,1.87)**	1.48 (1.05,2.08)***	4.07 (2.75,6.02)*
Model 4^d^	1 (ref)	1.32 (0.97,1.79)	1.53 (1.09,2.15)***	3.85 (2.60,5.71)*
**Cancer mortality**				
Death, no.	118	53	35	9
Model 1^a^	1 (ref)	1.35 (0.98,1.87)	1.88 (1.29,2.75)**	1.59 (0.81,3.13)
Model 2^b^	1 (ref)	1.39 (1.01,1.93)**	1.69 (1.15,2.49)**	1.54 (0.78,3.04)
Model 3^c^	1 (ref)	1.46 (1.05,2.03)**	1.47 (1.00,2.20)***	1.54 (0.78,3.06)
Model 4^d^	1 (ref)	1.47 (1.05,2.06)**	1.49 (1.00,2.22)***	1.56 (0.78,3.11)

CI, cognitive impairment; DM, diabetes mellitus; CVD, cardiovascular disease; HRs, hazard ratios.

a An unadjusted model.

b Adjusted for age and gender.

c Further adjusted for residence, education, marriage, smoking status, alcohol drinking, exercise, and BMI.

d Further adjusted for WC, chronic diseases (hypertension, dyslipidemia, coronary disease, COPD, and tumor), TC, TG, and uric acid.

*p<0.001;**p<0.01;***p ≤ 0.05.

**Figure 1:**
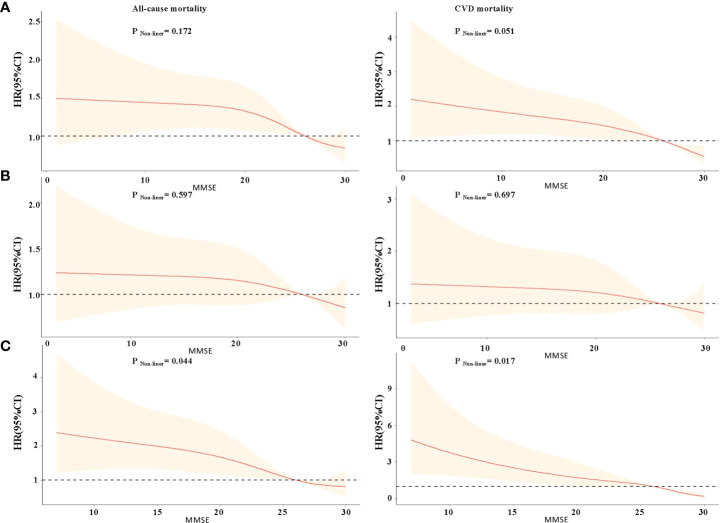
Restricted cubic splines for the association of MMSE score with all-cause and CVD mortality according to diabetes mellitus. **(A)** Total study participants; **(B)** non-diabetes mellitus group; **(C)** diabetes mellitus group. Hazard ratios (HRs) are indicated by red solid lines and 95% CIs by shaded areas. All models were adjusted for age, gender, residence, education, marriage, smoking status, alcohol drinking, exercise, BMI, WC, hypertension, dyslipidemia, coronary disease, COPD, tumor, TC, TG, and uric acid.

The restricted cubic splines of HRs for MMSE score are shown in **Figure 1A**. There was a linear inverse association between MMSE score and all-cause, CVD mortality in the total sample, but the inverse correlation association between MMSE score and all-cause mortality was not statistically significant when MMSE score <6 (p for non-linear >0.05). The shape of the curve for the association between MMSE score and mortality was significantly modified by DM. For participants without diabetes, there was a steep decrease in the risk of all-cause and CVD mortality with MMSE score <26, but the inverse correlation was not statistically significant (all p for non-linearity >0.05, **Figure 1B**). For participants with diabetes, it showed a nearly reverse J-shaped relationship; mortality risk was increased primarily among individuals with MMSE scores <26 (p for non-linear <0.05, **Figure 1C**).

### Interaction of diabetes and cognitive impairment on mortality

In the non-DM group, compared with normal cognition (reference group), having a CI was associated with an increased risk of all-cause mortality (HRs, 1.44; 95% CIs, 1.13,1.83), CVD mortality (HRs, 1.48; 95% CIs, 1.04,2.11), and cancer mortality (HRs, 1.51; 95% CIs, 1.01,2.28) after adjusting for all collected confounding variables. In addition, in the diabetes group, we observed that the estimated risk of all-cause and CVD mortality were significantly stronger in participants with CI than in the reference group (**Table 3**).

**Table 3:** Hazard ratios for the association between CI with all-cause and cause-specific mortality by DM or not.

	Non-DM		DM		Interaction^†^
	Normal cognition	Cognitive impairment	Normal cognition	Cognitive impairment
Participants, no.	2,836	587	900	176	
**All-cause mortality**					
Death, no.	346	108	156	57	
Model 1^a^	1(ref)	1.64(1.31,2.06)*	1(ref)	2.70(1.99,3.67)*	2.77(2.12,3.61)*
Model 2^b^	1(ref)	1.64(1.31,2.06)*	1(ref)	2.31(1.69,3.14)*	2.55(1.95,3.32)*
Model 3^c^	1(ref)	1.42(1.12,1.81)**	1(ref)	2.10(1.52,2.90)*	2.52(1.93,3.30)*
Model 4^d^	1(ref)	1.44(1.13,1.82)**	1(ref)	2.09(1.51,2.89)*	2.46(1.87,3.22)*
**CVD mortality**					
Death, no.	144	52	63	33	
Model 1^a^	1(ref)	2.52(1.82,3.48)*	1(ref)	3.93(2.57,6.03)*	3.87(2.72,5.52)*
Model 2^b^	1(ref)	1.79(1.28,2.49)**	1(ref)	3.29(2.14,5.06)*	3.48(2.44,4.97)*
Model 3^c^	1(ref)	1.45(1.02,2.06)***	1(ref)	3.08(1.97,4.85)*	3.32(2.31,4.77)*
Model 4^d^	1(ref)	1.48(1.04,2.11)***	1(ref)	3.16(2.02,5.05)*	3.15(2.19,4.55)*
**Cancer mortality**					
Death, no.	118	35	53	9	
Model 1^a^	1(ref)	1.83(1.25,2.68)**	1(ref)	1.25(0.62,2.55)	1.19(0.61,2.32)
Model 2^b^	1(ref)	1.67(1.13,2.47)**	1(ref)	1.17(0.57,2.40)	1.16(0.59,2.26)
Model 3^c^	1(ref)	1.50(1.00,2.26)***	1(ref)	1.03(0.50,2.16)	1.14(0.58,2.24)
Model 4^d^	1(ref)	1.51(1.01,2.28)***	1(ref)	1.04(0.50,2.16)	1.15(0.59,2.26)

CI, cognitive impairment; DM, diabetes mellitus; CVD, cardiovascular disease; HRs, hazard ratios.

a An unadjusted model.

b Adjusted for age and gender.

c Further adjusted for residence, education, marriage, smoking status, alcohol drinking, exercise, BMI.

d Further adjusted for WC, chronic diseases (hypertension, dyslipidemia, coronary disease, COPD, and tumor), TC, TG, uric acid.

*p<0.001; **p<0.01; ***p ≤ 0.05. ^†^Cognitive function status ∗Diabetes category.

The analysis of the fully adjusted Cox proportional hazard models that included an interaction term between DM and CI suggested a multiplicative negative interaction between DM and CI regarding the hazard of all-cause (HRs, 2.46; 95% CI, 1.87,3.22) and CVD mortality (HRs, 3.15 95% CI, 2.19,4.55) (p < 0.001, **Table 3**). The risk of participants with coexisting DM and CI on all-cause and CVD mortality is higher than the combined risk of death from one disease and two diseases alone.

### Stratified and sensitivity analysis

**Figure 2:**
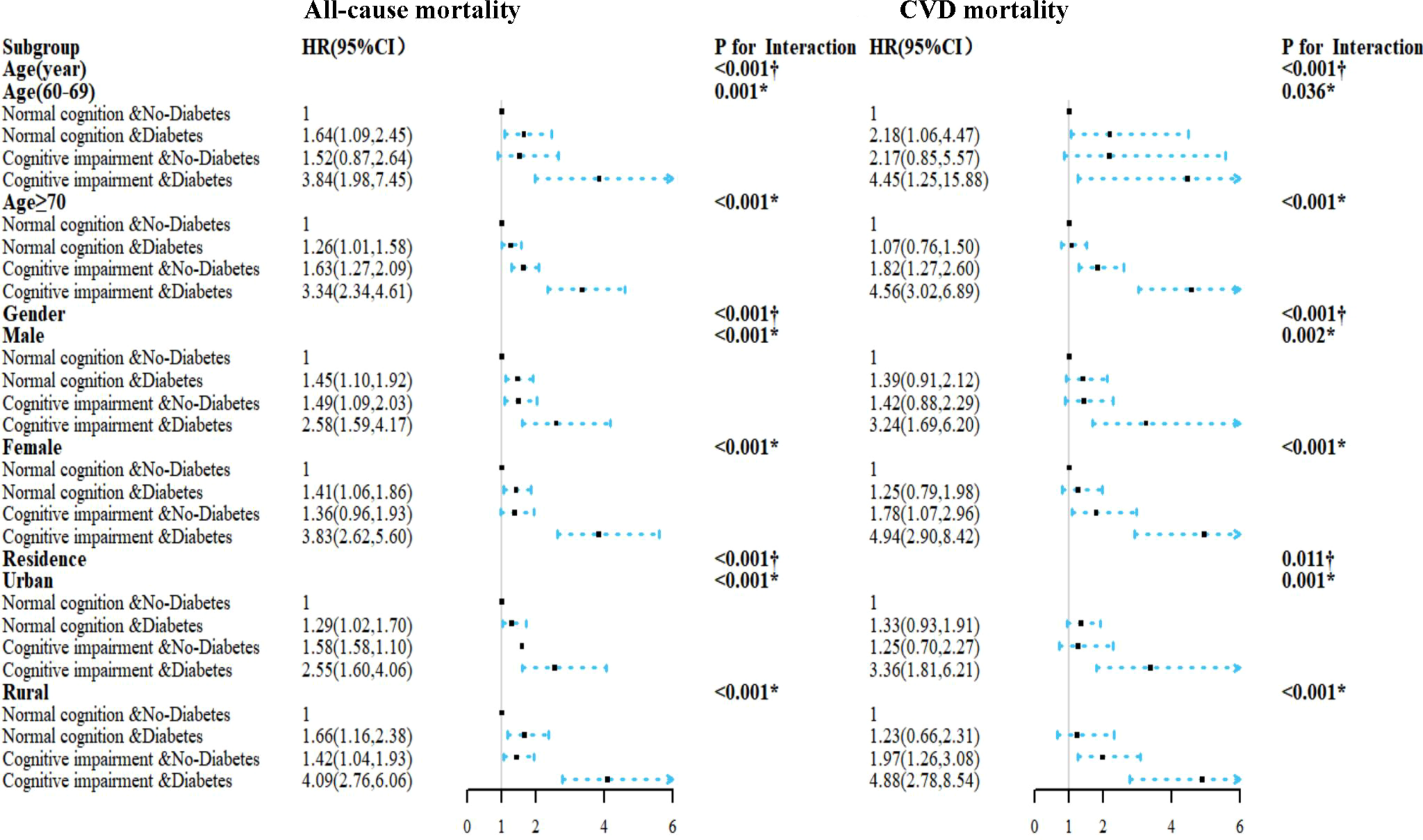
HRs for the combined associations of cognition impairment and diabetes mellitus with all-cause and CVD mortality according to the classification of age, gender, and residence. All models were adjusted for age (not in the age subgroup) and gender (not in the gender subgroup), residence (not in the residence subgroup), education, marriage, smoking status, alcohol drinking, exercise, BMI, WC, chronic diseases (hypertension, dyslipidemia, coronary disease, COPD, and tumor), TC, TG, and uric acid. The estimated HRs for each group are compared with normal cognition and non-diabetes groups. ^†^Interaction between the four-level joint variable of CI/DM and age, gender, or residence subgroup on mortality. *Interaction between diabetes mellitus and cognitive impairment on mortality.

The consistent results stratified by age, gender, and residence are displayed in **Figure 2**. Compared to other groups, the combination of DM and CI was associated with the highest risk of all-cause, CVD mortality in the fully adjusted model. Interestingly, the estimated risk of all-cause mortality was higher in participants aged 60–69 years than in participants aged ≥70 years, but the CVD mortality was lower in participants aged ≥70 years (all p for interaction <0.05). The association between diabetes, cognitive function, and mortality had gender/residence between-group differences (p for interaction < 0.05).

Regarding sensitivity analyses, the results were essentially unchanged when excluding participants who died within 2 years (**Supplementary eTable S1**), excluding participants with four kinds of self-reported diseases (**Supplementary eTable S2**), excluding participants with prediabetes (**Supplementary eTable S4**), or excluding participants with newly diagnosed diabetes (**Supplementary eTable S5**). The trend of overall effect was unchanged largely when additionally adjusting for family history of diseases (**Supplementary eTable 3**). Moreover, similar findings were found when using MMSE<23 cutoff or redefining CI (**Supplementary eTables S6**, **S7**). Using the new criteria of the severity of CI attenuated associations for the primary outcomes (**Supplementary eTables S8**, **S9**).

## Discussion

In this large prospective cohort study, we evaluated the combined effects of DM and CI on the risk of mortality. We also investigated whether the association between CI and mortality is potentially modified by DM. After adjusting for potential risk factors, participants with DM and CI had the highest risk of all-cause and CVD mortality, although the results did not reach statistical significance between CI, DM, and cancer mortality, which could be largely due to reduced power. Notably, the association between CI and mortality risk differs among older adults, which is greatly altered by patients with diabetes, and the association between DM, CI, and mortality was stronger among relatively younger old adults (aged 60–69 years). This study proved a multiplicative interaction between DM and CI with respect to all-cause and CV mortality in Chinese older adults and indicated that assessing a combination of DM and CI than a separate entity to predict the risk of mortality among older adults.

Our finding was that increased mortality risk was associated with CI and DM in older adults, which is consistent with those of previous observations (30–32). An Australia cohort study of 11,140 patients with diabetes focused on adults (≥55 years) demonstrated that CI was associated with an increased risk of all-cause and cardiovascular mortality (30). Another study of 559 participants with diabetes (aged ≥ 70 years) included in America found that older adults with diabetes and low levels of cognition were approximately 20% more likely to die than those with higher levels of cognition over a 2-year period (32). Two waves of the National Health and Nutrition Examination Survey in America displayed that cognitive impairment concomitant with other systemic vascular comorbidities predicted further increased risks of mortality, and HRs for all-cause mortality among participants with cognitive impairment concomitant with DM are as high as 2.24 (33), Our results are consistent with previously reported study. To our knowledge, this study is the first to reveal that DM and CI have a synergistic effect in increasing the risk of all-cause mortality, but our study found that the risk of all-cause mortality among individuals with DM and CI was higher than the results of the above study (33). Three main reasons exist: first, the two studies included different populations and races, and there were significant differences in the timing of the baseline surveys of these two studies, with the US study being conducted 20 years ago, while our study was conducted 10 years ago. At present, CVD is the leading cause of death in China, being the cause of 36.0% of deaths in the Chinese population, which may affect the value of the hazard ratios. Second, the cognitive screening scale adopted in those two studies was different. Third, much of the increased risk may be explained by the higher prevalence of chronic diseases (such as cardiovascular risk factors) among patients with CI. But even after controlling for these risk factors, the link between CI and death outcomes still had statistical significance in our study.

However, none of these previous studies considered whether the association between CI and mortality could be altered by DM. In our subgroup analysis of diabetes, we found that the risk of all-cause and CVD death in the DM group was much higher than that in the non-DM group after adjusting for potential confounders. We also found a linear (dose–response) relationship between MMSE and mortality, which was consistent with previous studies (25). Interestingly, we found that the shape of the curve depicting the association between MMSE and mortality differed according to DM. Although there is a non-linear relationship between MMSE with mortality in diabetic patients, it is most likely due to extreme values. Our results provide evidence that supports screening for CI and DM in older adults. Furthermore, several previous studies have explored the interaction effects of CI and age, gender, anemia, and BMI on mortality risk in different populations (25, 34). The present study expanded on the evidence, finding a significant interaction of DM and CI on all-cause and CVD mortality. These results may update policy development around health promotion and disease prevention in Chinese older adults.

Given the age difference in DM and CI, the analyses were stratified by age groups (60_69/≥70), and significant associations were observed in each subgroup, but the association between CI, DM, and the risk of all-cause and CVD mortality seemed stronger in younger participants (aged 60–69 years) than in older ones (aged 70 years or older). This may be due to “survivor bias,” which is commonly called selection bias due to loss to follow-up and can distort study results in geriatric populations (35, 36) or participants (aged ≥70 years) may represent the relatively healthier group of individuals. This association also had a gender-specific between-group difference. One potential explanation for this finding might be that causes and underlying diseases of DM and CI may differ by gender. Similarly, there are gender differences between CI, DM, and the risk of all-cause and CVD mortality. This may be associated with diet structure, chronic diseases, and higher levels of economic and medical in urban areas than in rural areas, resulting in a lower risk of mortality in urban areas. It is necessary to further explore the different patterns of mortality and possible influencing factors of CI in different regions.

The association between DM and CI was noticed by Miles et al. in 1922 (37). At present, the precise physiological pathways linking the two conditions remain largely undetermined, but they likely share common pathophysiological mechanisms, (38–41). (1) Anaerobic metabolism caused by sustained hyperglycemia damages brain cells and hypoglycemia disturb brain energy metabolism, leading to neuronal degeneration and hippocampal atrophy. (2) Abnormalities in the insulin signaling pathway can reduce brain insulin levels and insulin activity, affecting β-amyloid synthesis and breakdown and causing phosphorylated tau, which results in neuronal decline. (3) There are various other hypotheses, including altered cerebral blood flow, increased inflammatory mediators, and immune dysregulation. These biological changes further lead to impaired neuronal cell structure and function, thereby leading to CI.

Both DM and CI in combination could be categorized as an entity called “diabetic cognitive dysfunction,” which predicts the risk of mortality. The cause pathways underlying the observed association between CI, DM, and the risk of mortality remain to be elucidated, which means that the relationship between CI, DM, and mortality risk is complex and likely mediated by several other factors, such as characteristic factors of diabetes (chronic hyperglycemia, glucose fluctuations, and microvascular complications) and factors with associated diabetes (obesity and hypertension). DM combined with CI might leads to increased risks of falls and accidents (42), which might account for the high risks of mortality observed in our study. In addition, the increased risk of death in participants with both DM and CI might be mediated by CVD or cerebrovascular diseases. Cerebrovascular diseases are a significantly more common cause of death in vascular or mixed dementia patients (43). Abnormal levels of blood glucose could lead to advanced cerebrovascular diseases or CVD due to poor management or improper monitoring of DM in patients with CI (44). Notably, executive dysfunction and deficits in other cognitive domains could have negative impacts on the acquisition of disease self-management skills (45).

### Strengths and limitations

Several valuable strengths of the current study were as follows. First, this study was based on a prospective study design, used a relatively large sample size, and used a representative sample from urban and rural areas, which facilitate the generalization of findings. Second, many potential confounders were considered, and different adjustment strategies were presented to ensure the authenticity of the results. To the best of our knowledge, this is the first study to investigate the combined role of diabetes and CI in predicting mortality based on a cohort of old adults in community-dwelling Chinese older adults.

There are some limitations as well. First, only two glycemic indexes for the diagnosis of diabetes—FPG and HbA1c concentrations—were obtained; diagnostic criteria for diabetes did not include the oral glucose tolerance test (OGTT) in our study, which may result in misdiagnosis. However, after newly diagnosed diabetes patients were excluded, the results relying on self-reported diabetes remained stable. Moreover, our follow-up did not include newly diagnosed diabetes, and we will further explore whether the longitudinal changes are related to the increased risk of mortality in the future. Second, despite controlling for many covariates, residual confounders could not be completely eliminated due to unmeasured factors and unknown covariates such as a positive family history of CV diseases, obesity, and chronic renal, which may affect our results. However, comprehensive adjustment strategies were carried out, and the results were still robust. Third, we did not have information on the length of diabetes and diabetic complication, but the risk of microvascular and macrovascular diseases will increase over time, resulting in death. However, studies have shown that the length of diabetes was not related to mortality (29), and the results remained significant when further adjusting for the self-reported chronic disease. Finally, the data were based on participants from one province of China; hence, results may not necessarily be generalizable to other populations in China.

## Conclusion

In conclusion, this study highlighted the sizeable impact of co-occurring diabetes and cognitive impairment on mortality in general older adults. In addition, combined effects of cognitive impairment and diabetes further synergistically predicted greater risks of all-cause and CVD mortality than the sum of their independent effects. For the management of diabetic cognitive dysfunction, using brief cognitive screening measures as indicators of mortality for underlying sub-clinical and clinical diseases may yield significant benefits to older adults with diabetes as a means of improving survival.

## Data availability statement

The raw data supporting the conclusions of this article will be made available by the authors, without undue reservation.

## Ethics statement

The studies involving human participants were reviewed and approved by The Ethics Committee of Chinese PLA General Hospital. The patients/participants provided their written informed consent to participate in this study.

## Author contributions

ZL, SW, ML, YH, and YW conceived of the study design. ZL and SW conducted analyses. ZL, SW, SL and wrote the first draft of the article. XG, SL, YDW, DW, MY, and SC managed the data and provided help in the data analysis. RL, HL, XL, RJ, JG, and JW, make corrections and suggestions on the defects of the first draft of the article. YH and YW is the guarantor of this work and, as such, had full access to all the data in the study and takes responsibility for the integrity of the data and the accuracy of the data analysis. All authors contributed to the study design, critically reviewed draft versions and provided important intellectual content during revisions, and approved the final version of the manuscript.

## Funding

The study was supported by the Program of National Natural Science Foundation of China (Serial No.: 82173589,82173590) and Special Grant for the Prevention and Control of Infectious Diseases (2018ZX10713003).

## Acknowledgments

We sincerely thank those who participated in data collection and management.

## Conflict of interest

The authors declare that the research was conducted in the absence of any commercial or financial relationships that could be construed as a potential conflict of interest.

## Publisher’s note

All claims expressed in this article are solely those of the authors and do not necessarily represent those of their affiliated organizations, or those of the publisher, the editors and the reviewers. Any product that may be evaluated in this article, or claim that may be made by its manufacturer, is not guaranteed or endorsed by the publisher.
