# Supplementary material for: Synergistic impact of diabetes and cognitive impairment on all-cause and cause-specific mortality in Chinese older adults: A prospective population-based cohort study
Source: Front Endocrinol (Lausanne). 2022 Nov 14;13:997260. doi: 10.3389/fendo.2022.997260 (PMC9702801; doi:10.3389/fendo.2022.997260)
Supplement: Supplementary file 1 [file DataSheet_1.docx]

Supplemental Online Content

**eFigure1.Kaplan-Meier survival curves by the 4-level joint Diabetes/Cognition function groups in Chinese elderly.**

**eTable1. HRs(95%CIs) for All-Cause and Cause-Specific Mortality by DM and CI After Excluding Participants who Died Within 2 Years of Follow-up**

**eTable2.HRs (95%CIs) for All-Cause and Cause-Specific Mortality by DM and CI After Excluding Participants with 4 kinds of self-reported diseases**

**eTable3.HRs (95% CIs) for All-Cause and Cause-Specific Mortality by DM and CI additionally adjusting for family history of diseases**

**eTable4.HRs (95% CIs) for All-Cause and Cause-Specific Mortality by DM and CI After Excluding Participants with Prediabetes**

**eTable5.HRs (95% CIs) for All-Cause and Cause-Specific Mortality by DM and CI After Excluding Participants with newly diagnosed DM**

**eTable6.HRs (95% CIs) for All-Cause and Cause-Specific Mortality by DM and CI When Using MMSE< 23 Cutoff**

**eTable7.HRs (95% CIs) for All-Cause and Cause-Specific Mortality by DM and CI When Using MMSE Cutoff(<18, <21, <25)**

**eTable8. Hazard ratios for the Combined Associations of DM and CI with All-Cause and Cause-Specific Mortality**

**eTable9. Hazard ratios for the association between the severity of CI with all-cause and cause-specific mortality by DM or not**

**efigure 1. Kaplan-Meier survival curves by the 4-level joint Diabetes/Cognition function groups in Chinese elderly. (A)All-cause mortality;(B)CVD mortality**

| **eTable 1 .HRs (95% CIs) for All-Cause and Cause-Specific Mortality by DM and CI After Excluding Participants Died Within 2 Years of Follow-up** | | | | |
| --- | --- | --- | --- | --- |
| Group | Death,No. | Model 1 | Model 2 | Model 3 |
|  |  | HR (95% CI) | HR (95% CI) | HR (95% CI) |
| **All-cause mortality** | | | | |
| Normal cognition &Non-DM(n=2792)  Normal cognition &DM(n=886)  CI &Non-DM(n=562)  CI&DM(n=164) | 303  142  83  45 | 1  1.433(1.174,1.750)*  1.494(1.164,1.919)**  3.035(2.215,4.159)* | 1  1.431(1.168,1.753)**  1.327(1.025,1.717)***  3.011(2.187,4.145)* | 1  1.421(1.156,1.746)**  1.331(1.028,1.723)***  3.002(2.178,4.137)* |
| **CVD mortality** | | | | |
| Normal cognition &Non-DM(n=2792)  Normal cognition &DM(n=886)  CI &Non-DM(n=562)  CI&DM(n=164) | 132  58  42  27 | 1  1.354(0.994,1.845)  1.648(1.151,2.361)**  4.154(2.738,6.304)* | 1  1.371(1.001,1.878)***  1.418(0.979,2.055)  3.917(2.557,6.001)* | 1  1.281(0.931,1.761)  1.464(1.009,2.123)***  3.824(2.494,5.863)* |
| **Cancer mortality** | | | | |
| Normal cognition &Non-DM(n=2792)  Normal cognition &DM(n=886)  CI &Non-DM(n=562)  CI&DM(n=164) | 96  49  23  5 | 1  1.559(1.104,2.201)***  1.460(0.916,2.325)  1.108(0.450,2.727) | 1  1.553(1.093,2.205)***  1.389(0.861,2.240)  1.133(0.458,2.803) | 1  1.600(1.122,2.282)**  1.381(0.856,2.228)  1.159(0.468,2.870) |
| Abbreviations: CI, cognitive impairment; DM, diabetes mellitus; CVD, cardiovascular disease; HRs, hazard ratios.  a An unadjusted model;  b Adjusted for age and gender.  c Further adjusted for residence, education, marriage, smoking status, alcohol drinking, exercise, BMI  d Further adjusted for WC, chronic diseases (hypertension, dyslipidemia, coronary disease, COPD, and tumor ), TC, TG, uric acid  *P<0.001;**P<0.05 | | | | |

| **eTable 2.HRs (95% CIs) for All-Cause and Cause-Specific Mortality by DM and CI After Excluding Participants with 4 kinds of self-reported diseases** | | | | |
| --- | --- | --- | --- | --- |
| Group | Death, No. | Model 1 | Model 2 | Model 3 |
|  |  | HR (95% CI) | HR (95% CI) | HR (95% CI) |
| **All-cause mortality** | | | | |
| Normal cognition &Non-DM(n=816)  Normal cognition &DM(n=171）  CI&Non-DM（n=133）  CI&DM(n=35) | 77  19  24  10 | 1  1.128(0.683,1.864)  1.657(1.023,2.685)***  4.004(2.051,7.818)* | 1  1.180(0.707,1.967)  1.557(0.945,2.5660  3.752(1.861,7.568)* | 1  1.490(0.936,2.372)  1.675(1.053,2.664)***  3.990(2.009,7.924)* |
| **CVD mortality** | | | | |
| Normal cognition &Non-DM(n=816)  Normal cognition &DM(n=171）  CI&Non-DM（n=133）  CI&DM(n=35) | 22  3  9  4 | 1  0.610(0.183,2.041)  2.172(0.954,4.948)  6.747(2.250,20.227)* | 1  0.680(0.199,2.323)  2.164(0.930,5.039)  6.278(1.963,20.075)** | 1  0.941(0.347,2.557)  2.783(1.336,5.794)**  5.686(1.883,17.167)** |
| **Cancer mortality** | | | | |
| Normal cognition &Non-DM(n=816)  Normal cognition &DM(n=171）  CI&Non-DM（n=133）  CI&DM(n=35) | 32  11  8  3 | 1  1.598(0.805,3.171)  1.282(0.571,2.878)  2.515(0.762,8.302) | 1  1.856(0.924,3.726)  1.255(0.547,2.879)  3.038(0.878,10.516) | 1  2.099(1.085,4.062)***  1.318(0.599,2.901)  3.418(0.974,12.00) |
| Abbreviations: CI, cognitive impairment; DM, diabetes mellitus; CVD, cardiovascular disease; HR, hazard ratio.   \| **eTable 3.HRs (95% CIs) for All-Cause and Cause-Specific Mortality by DM and CI additionally adjusting for family history of hypertension, diabetes, and coronary disease** \| \| \| \| \| \| --- \| --- \| --- \| --- \| --- \| \| Group \| Death, No. \| Model 1 \| Model 2 \| Model 3 \| \| HR (95% CI) \| HR (95% CI) \| HR (95% CI) \| \| **All-cause mortality** \| \| \| \| \| \| Normal cognition &Non-DM(n=2836)  Normal cognition &DM(n=900)  CI&Non-DM(n=587)  CI&DM(n=176) \| 346  156  108  57 \| 1  1.39(1.15,1.68)*  1.63(1.30,2.03)*  3.21(2.42,4.26)* \| 1  1.42(1.16,1.72)*  1.39(1.09,1.74)**  3.08(2.30,4.11)* \| 1  1.352(1.105,1.653)*  1.265(1.012,1.581)***  2.644(1.983,3.525)* \| \| **CVD mortality** \| \| \| \| \| \| Normal cognition &Non-DM(n=2836)  Normal cognition &DM(n=900)  CI&Non-DM(n=587)  CI&DM(n=176) \| 144  63  52  33 \| 1  1.35(1.01,1.82)**  1.79(1.29,2.49)*  4.45(3.05,6.51)* \| 1  1.32(0.97,1.79)  1.53(1.09,2.15)***  3.85(2.60,5.71)* \| 1  1.276(0.931,1.748)  1.364(0.977,1.904)  3.451(2.327,5.118)* \| \| **Cancer mortality** \| \| \| \| \| \| Normal cognition &Non-DM(n=2836)  Normal cognition &DM(n=900)  CI&Non-DM(n=587)  CI&DM(n=176) \| 118  53  35  9 \| 1  1.39(1.01,1.93)**  1.69(1.15,2.49)**  1.54(0.78,3.04) \| 1  1.47(1.05,2.06)**  1.49(1.00,2.22)***  1.56(0.78,3.11) \| 1  1.276(0.931,1.748)  1.364(0.977,1.904)  3.451(2.327,5.118)* \| \| Abbreviations: CI, cognitive impairment; DM, diabetes mellitus; CVD, cardiovascular disease; HR, hazard ratio.  a Adjusted for age and gender.  b Further adjusted for residence, education, marriage, smoking status, alcohol drinking, exercise, BMI  c Further adjusted for WC, chronic diseases (hypertension, dyslipidemia, coronary disease, COPD, and tumor ),TC, TG, uric acid, and family history of hypertension, diabetes, and coronary heart disease  *P<0.001;**P<0.01;***P<0.05 \| \| \| \| \|   a Adjusted for age and gender.  b Further adjusted for residence, education, marriage, smoking status, alcohol drinking, exercise, BMI  c Further adjusted for WC, TC, TG, uric acid, chronic diseases (COPD)  *P<0.001;**P<0.01;***P<0.05 | | | | |

| **eTable4.HRs (95% CIs) for All-Cause and Cause-Specific Mortality by DM and CI After Excluding Participants with Prediabetes** | | | | |
| --- | --- | --- | --- | --- |
| Group | Death, No. | Model 1 | Model 2 | Model 3 |
|  |  | HR (95% CI) | HR (95% CI) | HR (95% CI) |
| **All-cause mortality** | | | | |
| Normal cognition &Non-DM(n=1228)  Normal cognition &DM(n=900)  CI&Non-DM(n=238)  CI&DM(n=176) | 140  156  44  57 | 1  1.339(1.065,1.684)***  1.660(1.173,2.350)**  3.077(2.254,4.201)* | 1  1.371(1.081,1.740)**  1.331(0.931,1.902)  3.006(2.181,4.143)* | 1  1.341(1.049,1.714)**  1.344(0.940,1.923)  2.912(2.108,4.022)* |
| **CVD mortality** | | | | |
| Normal cognition &Non-DM(n=1228)  Normal cognition &DM(n=900)  CI&Non-DM(n=238)  CI&DM(n=176) | 49  63  17  33 | 1  1.561(1.073,2.270)**  1.818(1.033,3.201)**  5.209(3.333,8.143)* | 1  1.536(1.042,2.265)***  1.535(0.859,2.743)  4.910(3.093,7.792)* | 1  1.413(0.946,2.110)  1.555(0.867,2.787)  4.553(2.862,7.244)* |
| **Cancer mortality** | | | | |
| Normal cognition &Non-DM(n=1228)  Normal cognition &DM(n=900)  CI&Non-DM(n=238)  CI&DM(n=176) | 50  53  16  9 | 1  1.272(0.863,1.876)  1.845(1.035,3.288)***  1.404(0.687,2.870) | 1  1.317(0.881,1.969)  1.557(0.860,2.822)  1.363(0.659,2.822) | 1  1.307(0.865,1.976)  1.534(0.845,2.783)  1.379(0.664,2.863) |
| Abbreviations: CI, cognitive impairment; DM, diabetes mellitus; CVD, cardiovascular disease; HR, hazard ratio.  a Adjusted for age and gender.  b Further adjusted for residence, education, marriage, smoking status, alcohol drinking, exercise, BMI  c Further adjusted for WC, chronic diseases (hypertension, dyslipidemia, coronary disease, COPD, and tumor ) ,TC, TG, uric acid  *P<0.001;**P<0.01;***P<0.05 | | | | |

| **eTable 5.HRs (95% CIs) for All-Cause and Cause-Specific Mortality by DM and CI after excluding participants with newly diagnosed DM** | | | | |
| --- | --- | --- | --- | --- |
| Group | Death, No. | Model 1 | Model 2 | Model 3 |
|  |  | HR (95% CI) | HR (95% CI) | HR (95% CI) |
| **All-cause mortality** | | | | |
| Normal cognition &Non-DM(n=2914)  Normal cognition &DM(n=822)  CI&Non-DM(n=596)  CI&DM(n=167) | 354  148  112  53 | 1  1.467(1.211,1.778)*  1.671(1.342,2.080)*  3.235(2.418,4.328)* | 1  1.492(1.229,1.813)*  1.418(1.130,1.778)**  3.156(2.349,4.240)* | 1  1.508(1.237,1.839)*  1.440(1.147,1.808)**  3.068(2.278,4.132)* |
| **CVD mortality** | | | | |
| Normal cognition &Non-DM(n=2914)  Normal cognition &DM(n=822)  CI&Non-DM(n=596)  CI&DM(n=167) | 146  61  54  31 | 1  1.470(1.090,1.983)**  1.866(1.350,2.578)*  4.559(3.081,6.744)* | 1  1.481(1.094,2.004)***  1.570(1.124,2.193)**  4.317(2.896,6.436)* | 1  1.420(1.042,1.935)***  1.616(1.154,2.262)**  3.969(2.651,5.941)* |
| **Cancer mortality** | | | | |
| Normal cognition &Non-DM(n=2914)  Normal cognition &DM(n=822)  CI&Non-DM(n=596)  CI&DM(n=167) | 122  49  37  7 | 1  1.418(1.017,1.976)***  1.748(1.198,2.549)***  1.284(0.598,2.757) | 1  1.470(1.050,2.057)***  1.553(1.052,2.293)***  1.278(0.592,2.758) | 1  1.522(1.081,2.143)***  1.552(1.050,2.295)***  1.295(0.598,2.805) |
| Abbreviations: CI, cognitive impairment; DM, diabetes mellitus; CVD, cardiovascular disease; HR, hazard ratio.  a Adjusted for age and gender.  b Further adjusted for residence, education, marriage, smoking status, alcohol drinking, exercise, BMI  c Further adjusted for WC, chronic diseases (hypertension, dyslipidemia, coronary disease, COPD, and tumor ), TC, TG, uric acid  *P<0.001;**P<0.01;***P<0.05 | | | | |

| **eTable 6.HRs (95% CIs) for All-Cause and Cause-Specific Mortality by DM and CI When using MMSE< 23 Cutoff** | | | | |
| --- | --- | --- | --- | --- |
| Group | Death, No. | Model 1 | Model 2 | Model 3 |
|  |  | HR (95% CI) | HR (95% CI) | HR (95% CI) |
| **All-cause mortality** | | | | |
| Normal cognition &Non-DM(n=2380)  Normal cognition &DM(n=1043)  CI&Non-DM(n=752)  CI&DM(n=324) | 292  162  127  86 | 1  1.342(1.089,1.653)**  1.649(1.348,2.018)*  3.046(2.383,3.894)* | 1  1.355(1.097,1.674)**  1.213(0.969,1.519)  2.439(1.881,3.162)* | 1  1.371(1.105,1.701)**  1.214(0.968,1.522)  2.382(1.831,3.099)* |
| **CVD mortality** | | | | |
| Normal cognition &Non-DM(n=2380)  Normal cognition &DM(n=1043)  CI&Non-DM(n=752)  CI&DM(n=324) | 114  82  48  48 | 1  1.304(0.930,1.828)  2.140(1.586,2.888)*  4.460(3.158,6.300)* | 1  1.312(0.932,1.846)  1.582(1.136,2.203)**  3.436(2.384,4.952)* | 1  1.246(0.879,1.765)  1.518(1.086,2.120)**  3.196(2.202,4.639)* |
| **Cancer mortality** | | | | |
| Normal cognition &Non-DM(n=2380)  Normal cognition &DM(n=1043)  CI&Non-DM(n=752)  CI&DM(n=324) | 109  44  50  12 | 1  1.419(1.015,1.985)***  1.223(0.850,1.760)  1.131(0.619,2.067) | 1  1.484(1.056,2.087)***  0.919(0.610,1.384)  0.972(0.521,1.816) | 1  1.553(1.098,2.196)**  0.919(0.609,1.388)  0.974(0.519,1.827) |
| Abbreviations: CI, cognitive impairment; DM, diabetes mellitus; CVD, cardiovascular disease; HR, hazard ratio.  a Adjusted for age, and gender.  b Further adjusted for residence, education, marriage, smoking status, alcohol drinking, exercise, BMI  c Further adjusted for WC, chronic diseases (hypertension, dyslipidemia, coronary disease, COPD, and tumor ), TC, TG, uric acid  *P<0.001;**P<0.01;***P<0.05 | | | | |

| **eTable 7.HRs (95% CIs) for All-Cause and Cause-Specific Mortality by DM and CI When Using MMSE Cutoff(<18, <21, <25)** | | | | |
| --- | --- | --- | --- | --- |
| Group | Death, No. | Model 1 | Model 2 | Model 3 |
|  |  | HR (95% CI) | HR (95% CI) | HR (95% CI) |
| **All-cause mortality** | | | | |
| Normal cognition &Non-DM(n=2734)  Normal cognition &DM(n=883)  CI&Non-DM(n=689)  CI&DM(n=193) | 333  153  121  60 | 1  1.381(1.140,1.673)*  1.454(1.172,1.802)*  2.953(2.239,3.895)* | 1  1.418(1.167,1.723)*  1.268(1.015,1.583)***  2.883(2.177,3.818)* | 1  1.423(1.167,1.735)*  1.272(1.018,1.590)***  2.830(2.132,3.758)* |
| **CVD mortality** | | | | |
| Normal cognition &Non-DM(n=2734)  Normal cognition &DM(n=883)  CI&Non-DM(n=689)  CI&DM(n=193) | 139  61  57  35 | 1  1.317(0.975,1.781)  1.532(1.113,2.111)**  4.054(2.790,5.892)* | 1  1.357(0.999,1.843)  1.350(0.969,1.880)  3.809(2.598,5.585)* | 1  1.296(0.949,1.770)  1.381(0.990,1.926)  3.617(2.457,5.325)* |
| **Cancer mortality** | | | | |
| Normal cognition &Non-DM(n=2734)  Normal cognition &DM(n=883)  CI&Non-DM(n=689)  CI&DM(n=193) | 116  53  37  9 | 1  1.387(1.002,1.921)***  1.414(0.968,2.067)  1.330(0.673,2.627) | 1  1.452(1.043,2.022)***  1.244(0.841,1.839)  1.306(0.658,2.591) | 1  1.500(1.072,2.100)***  1.239(0.838,1.834)  1.335(0.671,2.658) |
| Abbreviations: CI, cognitive impairment; DM, diabetes mellitus; CVD, cardiovascular disease; HR, hazard ratio.  a Adjusted for age and gender.  b Further adjusted for residence, education, marriage, smoking status, alcohol drinking, exercise, BMI  c Further adjusted for WC, chronic diseases (hypertension, dyslipidemia, coronary disease, COPD, and tumor ), TC , TG, uric acid  *P<0.001;**P<0.01;***P<0.05 | | | | |

| **eTable8 \|Hazard ratios for the Combined Associations of DM and CI with All-Cause and Cause-Specific Mortality** | | | | | |
| --- | --- | --- | --- | --- | --- |
|  | Death, No. | **Model 1** | **Model 2** | **Model3** | **Model4** |
| **All-cause mortality** | | | | | |
| **Normal cognition** |  |  |  |  |  |
| Non-Diabetes(n=2180) | 264 | 1(ref) | 1(ref) | 1(ref) | 1(ref) |
| Diabetes(n=846) | **119** | 1.699(1.364,2.117)* | 1.592(1.276,1.985)* | 1.237(0.977,1.565) | 1.260(0.996,1.594) |
| **Mild Cognitive impairment** |  |  |  |  |  |
| Non-Diabetes(n=397) | 71 | 2.690(2.056,3.519)* | 1.889(1.430,2.494)* | 1.277(0.934,1.747) | 1.312(0.958,1.797) |
| Diabetes(n=695) | 115 | 1.285(1.032,1.600)** | 1.317(1.057,1.639)** | 1.328(1.064,1.658)** | 1.337(1.066,1.676)** |
| **Severe Cognitive impairment** |  |  |  |  |  |
| Non-Diabetes(n=273) | **65** | 2.777(2.113,3.648)* | 2.845(2.161,3.745)* | 2.268(1.701,3.024)* | 2.313(1.729,3.094)* |
| Diabetes(n=108) | 33 | 4.557(3.162,6.567)* | 3.883(2.683,5.621)* | 3.126(2.123,4.602)* | 3.004(2.037,4.430)* |
| **CVD mortality** | | | | | |
| **Normal cognition** |  |  |  |  |  |
| Non-Diabetes(n=2180) | **102** | 1(ref) | 1(ref) | 1(ref) | 1(ref) |
| Diabetes(n=846) | 57 | 2.251(1.618,3.131)* | 2.037(1.462,2.837)* | 1.550(1.092,2.202)** | 1.561(1.099,2.219)** |
| **Mild Cognitive impairment** |  |  |  |  |  |
| Non-Diabetes(n=397) | 37 | 4.031(2.732,5.949)* | 2.493(1.665,3.731)* | 1.779(1.131,2.799)** | 1.823(,1.155,2.879)** |
| Diabetes(n=695) | 42 | 1.208(0.843,1.730) | 1.249(0.871,1.789) | 1.246(0.867,1.793) | 1.164(0.803,1.686) |
| **Severe Cognitive impairment** |  |  |  |  |  |
| Non-Diabetes(n=273) | **34** | 3.963(2.680,5.861)* | 4.024(2.713,5.967)* | 3.156(2.089,4.769)* | 3.151(2.074,4.788)* |
| Diabetes(n=108) | **20** | 7.786(4.787,12.663)* | 6.133(3.749,10.034)* | 4.719(2.812,7.918)* | 4.374(2.601,7.356)* |
| Abbreviations: CI, cognitive impairment; DM, diabetes mellitus; CVD, cardiovascular disease; HRs, hazard ratios.  a An unadjusted model;  b Adjusted for age and gender.  c Further adjusted for residence, education, marriage, smoking status, alcohol drinking, exercise, BMI  d Further adjusted for WC, chronic diseases (hypertension, dyslipidemia, coronary disease, COPD, and tumor ),TC, TG, uric acid  *P<0.001;**P<0.05 | | | | | |

| **eTable 9 \| Hazard ratios for the association between the severity of CI with all-cause and cause-specific mortality by DM or not** | | | | | | | |
| --- | --- | --- | --- | --- | --- | --- | --- |
|  | Non-DM | | | DM | | | P for interaction |
|  | Normal cognition | Mild Cognitive impairment | Severe Cognitive impairment | Normal cognition | Mild Cognitive impairment | Severe Cognitive impairment |  |
| Participants | 2180 | 846 | 397 | 695 | 273 | 108 |  |
| All-cause | | | | | | | |
| Death,No. | 264 | 119 | 71 | 115 | 65 | 33 |  |
| Model 1^a^ | 1(ref) | 1.60(1.28,1.99)* | 1.90(1.43,2.53)* | 1(ref) | 2.14(1.57,2.93)* | 2.91(1.93,4.39)* |  |
| Model 2^b^ | 1(ref) | 1.21(0.95,1.54) | 1.29(0.92,1.80) | 1(ref) | 1.82(1.29,2.58)* | 2.40(1.52,3.79)* |  |
| Model 3^c^ | 1(ref) | 1.24(0.97,1.58) | 1.32(0.95,1.85) | 1(ref) | 1.85(1.30,2.63)* | 2.39(1.51,3.80)** | <0.001 |
| CVD | | | | | | | |
| Death,No. | 102 | 57 | 37 | 42 | 34 | 20 |  |
| Model 1^a^ | 1(ref) | 2.04(1.46,2.85)* | 2.46(1.62,3.72)* | 1(ref) | 3.21(2.02,5.10)* | 5.04(2.86,8.89)* |  |
| Model 2^b^ | 1(ref) | 1.41(0.98,2.03) | 1.56(0.96,2.54) | 1(ref) | 3.02(1.81,5.04)* | 5.00(2.66,9.39)* |  |
| Model 3^c^ | 1(ref) | 1.44(0.99,2.07) | 1.60(0.98,2.61) | 1(ref) | 3.28(1.94,5.54)* | 5.49(2.87,10.51)* | <0.001 |
| Abbreviations: CI, cognitive impairment; DM,diabetes mellitus; CVD, cardiovascular disease; HRs, hazard ratio.  a Adjusted for age, and gender .  b Further adjusted for residence, education, marriage, smoking status, alcohol drinking, exercise, BMI  c Further adjusted for WC, chronic diseases (hypertension, dyslipidemia, coronary disease, COPD, and tumor ) ,TC ,TG, uric acid  *P<0.001;**P<0.01;***P<0.05 | | | | | | | |
